# Chronological attenuation of NPRA/PKG/AMPK signaling promotes vascular aging and elevates blood pressure

**DOI:** 10.1111/acel.13699

**Published:** 2022-08-25

**Authors:** Changkun Long, Hongfei Liu, Wenxing Zhan, Liping Chen, Zhenping Yu, Shane Tian, Yang Xiang, Shenghan Chen, Xiao‐Li Tian

**Affiliations:** ^1^ Vascular Function Laboratory Human Aging Research Institute and School of Life Science, Nanchang university, and Jiangxi Key Laboratory of Human Aging Nanchang China; ^2^ Institute of Translational Medicine Nanchang University Nanchang China; ^3^ School of Life Science, Nanchang University Nanchang China; ^4^ Department of Biochemistry/Chemistry Ohio State University Columbus Ohio USA; ^5^ Metabolic Control and Aging Human Aging Research Institute and School of Life Science, Nanchang university, and Jiangxi Key Laboratory of Human Aging Nanchang China; ^6^ Aging and Vascular Diseases Human Aging Research Institute and School of Life Science, Nanchang university, and Jiangxi Key Laboratory of Human Aging Nanchang China

**Keywords:** AMPK, endothelial cell, NPRA, PKG, senescence

## Abstract

Hypertension is common in elderly population. We designed to search comprehensively for genes that are chronologically shifted in their expressions and to define their contributions to vascular aging and hypertension. RNA sequencing was conducted to search for senescence‐shifted transcripts in human umbilical vein endothelial cells (HUVECs). Small interfering RNA (siRNA), small‐molecule drugs, CRISPR/Cas9 techniques, and imaging were used to determine genes' function and contributions to age‐related phenotypes of the endothelial cell and blood vessel. Of 25 genes enriched in the term of “regulation of blood pressure,” *NPRA* was changed most significantly. The decreased *NPRA* expression was replicated in aortas of aged mice. The knockdown of *NPRA* promoted HUVEC senescence and it decreased expressions of protein kinase cGMP‐dependent 1 (PKG), sirtuin 1 (*SIRT1*), and endothelial nitric oxide synthase (*eNOS*). Suppression of *NPRA* also decreased the phosphorylation of AMP‐activated protein kinase (AMPK) as well as the ratio of oxidized nicotinamide adenine dinucleotide (NAD^+^)/reduced nicotinamide adenine dinucleotide (NADH) but increased the production of reactive oxygen species (ROS). 8‐Br‐cGMP (analog of cGMP), or AICAR (AMPK activator), counteracted the observed changes in HUVECs. The *Npr1* ^+/−^ mice presented an elevated systolic blood pressure and their vessels became insensitive to endothelial‐dependent vasodilators. Further, vessels from *Npr1* ^+/−^ mice increased *Cdkn1a* but decreased *eNos* expressions. These phenotypes were rescued by intravenously administrated 8‐Br‐cGMP and viral overexpression of human *PKG*, respectively. In conclusion, we demonstrate NPRA/PKG/AMPK as a novel and critical signaling axis in the modulation of endothelial cell senescence, vascular aging, and hypertension.

## Graphical abstract

Natriuretic peptide receptor A (NPRA) is chronologically decreased in senescent endothelial cell and aged vessels. The decrease in NPRA expression promotes endothelial cell senescence, vascular aging, and hypertension through NPRA/PKG/AMPK signaling axis in both endothelial cell and engineered animal models.

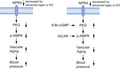

**Abbreviations**
Ach: acetylcholine
AMPK: AMP‐activated protein kinase
EVG: elastic van gieson
HUVECs: human umbilical vein endothelial cells
IL6: interleukin‐6
IL8: interleukin‐8
NAD^+^
: nicotinamide adenine dinucleotide
NO: nitric oxide
NPRA: natriuretic peptide receptor A
P21: cyclin‐dependent kinase inhibitor 1A
PDL: population doubling level
p‐eNOS: phosphorylated endothelial nitric oxide synthase
PKG: protein kinase cGMP‐dependent 1
ROS: reactive oxygen species
SASP: senescence‐associated secretory phenotype
SA‐β‐gal: senescence‐associated beta‐galactosidase
SIRT1: silencing information regulator 2 related enzyme 1
SNP: sodium nitroprusside

## 1 INTRODUCTION

Advanced age has been recognized as a critical risk factor for common forms of vascular diseases, such as hypertension (Collaboration, 2019), atherosclerotic diseases (Khan et al., 2012; Li et al., 2018), and aneurysms (Nordon et al., 2011). These cardiocerebral vascular diseases increase disability and remain as the top killer for the adult worldwide, despite of the decreased mortality rate found in developed countries recently (Mensah et al., 2017; Zhao et al., 2019). Hypertension remains the most common age‐related vascular disease, affecting 77.0% of adults above 65 years in USA (Virani et al., 2021) and 67.8% of those above 60 years in China (Ma et al., 2020; Xing et al., 2020), featured with isolated systolic hypertension (Chobanian, 2007), and it is well‐established a risk factor to many other life‐threatening cardiocerebral diseases (Bilen & Wenger, 2020; Cortese et al., 2020; D'Anci et al., 2020; Do et al., 2015; Doumas et al., 2017; Li & Lerman, 2020).

Numerous studies have demonstrated that a vascular aging positively correlates with the occurrence of hypertension and atherosclerotic plaque (Bochenek et al., 2016; Hammond & Rich, 2019; Harvey et al., 2015). Supportively, children with sign of early vascular aging exhibit elevated blood pressure (Litwin et al., 2016) and those who suffer from progeria are affected by atherosclerosis and hypertension (Rosman et al., 2001). However, how vascular aging contributes to these vascular conditions remains unknown.

As a vessel ages, it presents several features. For example, an elderly vessel has an increased intima‐media thickness in morphology (Homma et al., 2001). Functionally, the aged vessel becomes stiffed (Mitchell, 2021) and presents contractile particularly endothelium‐dependent contractile dysfunction. The expression of genes related to vascular functions, such as angiogenesis and contraction, are changed. In addition, an array of senescence‐associate markers, including those for cell cycle control (*CDKN1A* and *CDKN2A*) and inflammation (interleukins) are elevated. These alterations eventually increase the susceptibility to atherosclerosis and blood pressure elevation, which contribute to age‐related diseases and geriatric syndromes (Tian & Li, 2014).

NPRA (natriuretic peptide receptor A, also known as NPR1 or guanylyl cyclase A), is a transmembrane protein expressed in the vascular endothelium (Kuhn, 2016). Activation of NPRA requires a binding of natriuretic peptides NPPA (ANP) or NPPB (BNP) to its extracellular domain and this promotes the production of cyclic guanosine monophosphate (cGMP), which in turn activates protein kinase cGMP‐dependent 1 (PRKG1 or commonly used as PKG). We have reported that ANP and its processing enzyme CORIN decrease blood pressure (Chen et al., 2015) and null‐function of NPRA in the mouse model exhibits an elevated blood pressure, increased atherosclerosis, and inflammation (Oliver et al., 1997). All of these demonstrate that the NPRA‐mediated signaling cascade (NPRA/cGMP/PKG) is critical to maintain blood pressure (Song et al., 2015) and the homeostasis of endothelial cells (Tokudome et al., 2016). To date, no reports ever link NPRA/cGMP/PKG signaling to vascular aging.

In this study, we designed to search for transcriptionally changed genes during human endothelial cell senescence at genome level and evaluate their contributions to vascular aging and hypertension in mouse models, and at the end, we demonstrate that NPRA is decreased with aging, which not only elevates blood pressure but also promotes endothelial cell senescence and vascular aging through PKG‐AMP‐activated protein kinase (AMPK)‐associated signaling axis.

## 2 RESULTS

### 2.1 *NPRA* is decreased in senescent HUVEC and aged blood vessel

**FIGURE 1:**
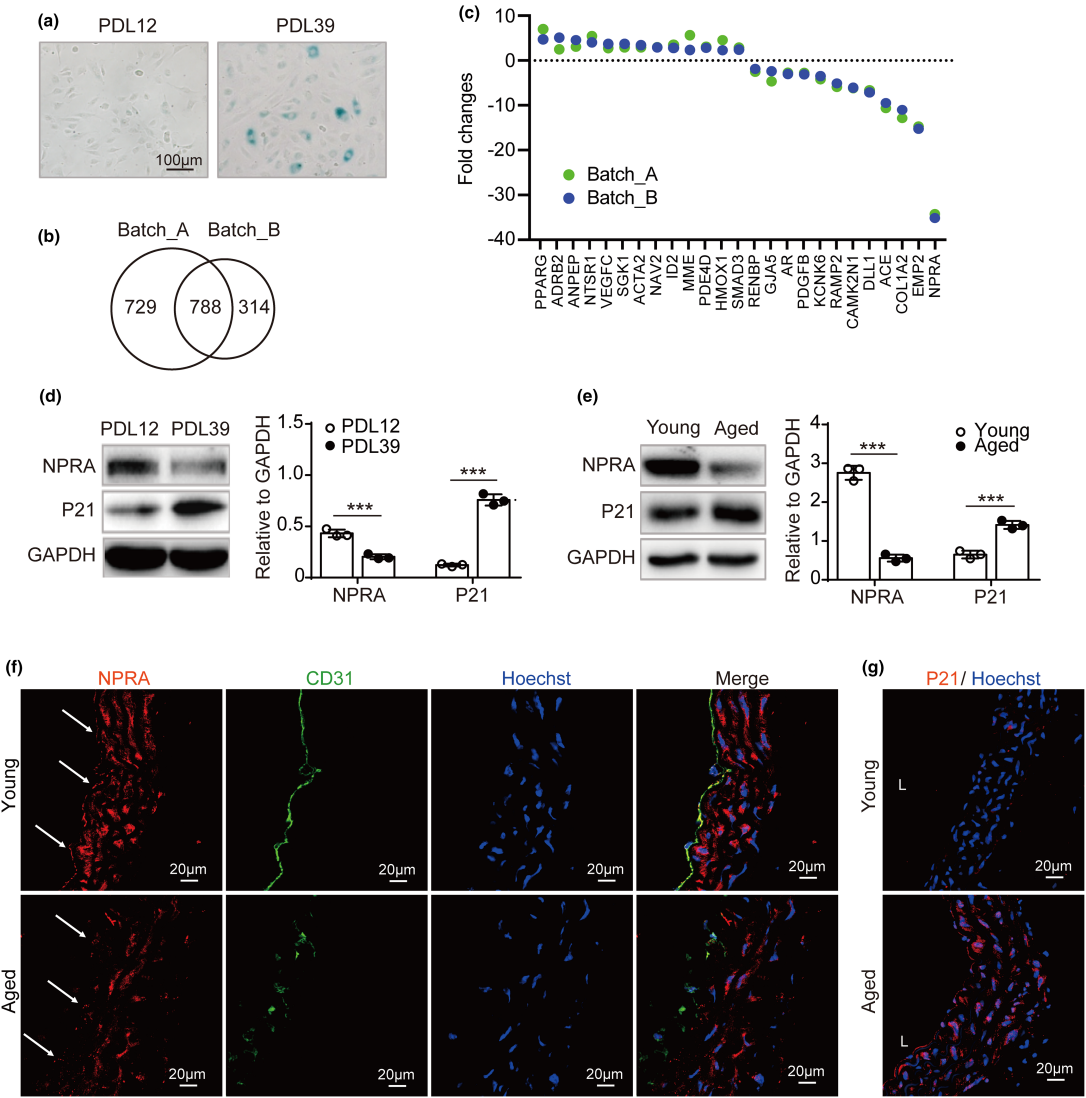
Decreased *NPRA* expression in HUVEC and aged blood vessel. (a) SA‐β‐gal staining for HUVECs at PDL 12 and PDL 39. (b) Overlapped 788 genes transcriptionally shifted from PDL 12 to 39 between two independent cord‐derived endothelial cells (Batch_A and Batch_B) by RNA sequencings. (c) Fold changes (PDL39 vs. 12) of the expression of 25 genes associated with term of “blood pressure regulation” in Batch_A (green) and Batch_B (blue). (d) Verification of NPRA and P21 expression at PDL 39 vs 12 by Western blot analysis. NPRA and P21 expression, normalized to GAPDH levels, based on the densitometry, respectively. Statistical significance analyzed using Student's *t*‐test. (e) Western blot for NPRA and P21 expression in thoracic aorta from young (4 months) and aged (14 months) mice (*n* = 3). Analysis of endogenous NPRA and P21 levels, normalized to GAPDH levels, by densitometry, respectively. Statistical comparison by Student's *t*‐test. (f) Expression of NPRA (red) and CD31 (green) in frozen aorta tissue sections from young (4 months) and aged (14 months) mice (*n* = 3). Nuclei (blue) stained by Hoechst. Arrows denote endothelium. (g) Immunofluorescence staining for, P21 (red) and nuclei (blue) with frozen aorta tissue sections from young (4 months) and aged (14 months) mice (*n* = 3). Values are mean ± SD. ***p < 0.001

Senescent cells exhibit a positive staining of SA‐β‐gal at pH 6.0 (Dimri et al., 1995). PDL 39 had significantly more SA‐β‐gal stained cells than PDL 12 (Figure 1a), indicative of successful replicative senescent model. Following removal of low abundant transcripts in HUVECs (RPKM <2), 788 genes were found to be transcriptionally shifted from PDL 12 to 39 in two independent cord‐derived endothelial cells and 25 were enriched under the term of “regulation of blood pressure” (Figure 1b). Of 25 genes, *NPRA* was decreased by 34‐fold from PDL 12 to 39, representing the most significantly shifted gene (Figure 1c). The decreased NPRA expression was verified in another batch of senescent endothelial cells (PDL 39 vs. 12) by Western blot, as seen in Figure 1d that, opposite to P21, the protein level of NPRA was decreased significantly in cells from PDL 39 compared with PDL 12. Moreover, we examined NPRA expression in thoracic aorta from 4‐month‐old (young) and 14‐month‐old (aged) mice by Western blot and immunofluorescence staining of the frozen aorta sections. As observed in Figure 1e–g, the signal for NPRA and P21 protein was inversely correlated in aorta with increased ages. Together, these experiments demonstrate that the expression of *NPRA* gene is chronologically prohibited with ages.

### 2.2 Knockdown of *NPRA* promotes endothelial cellular senescence

**FIGURE 2:**
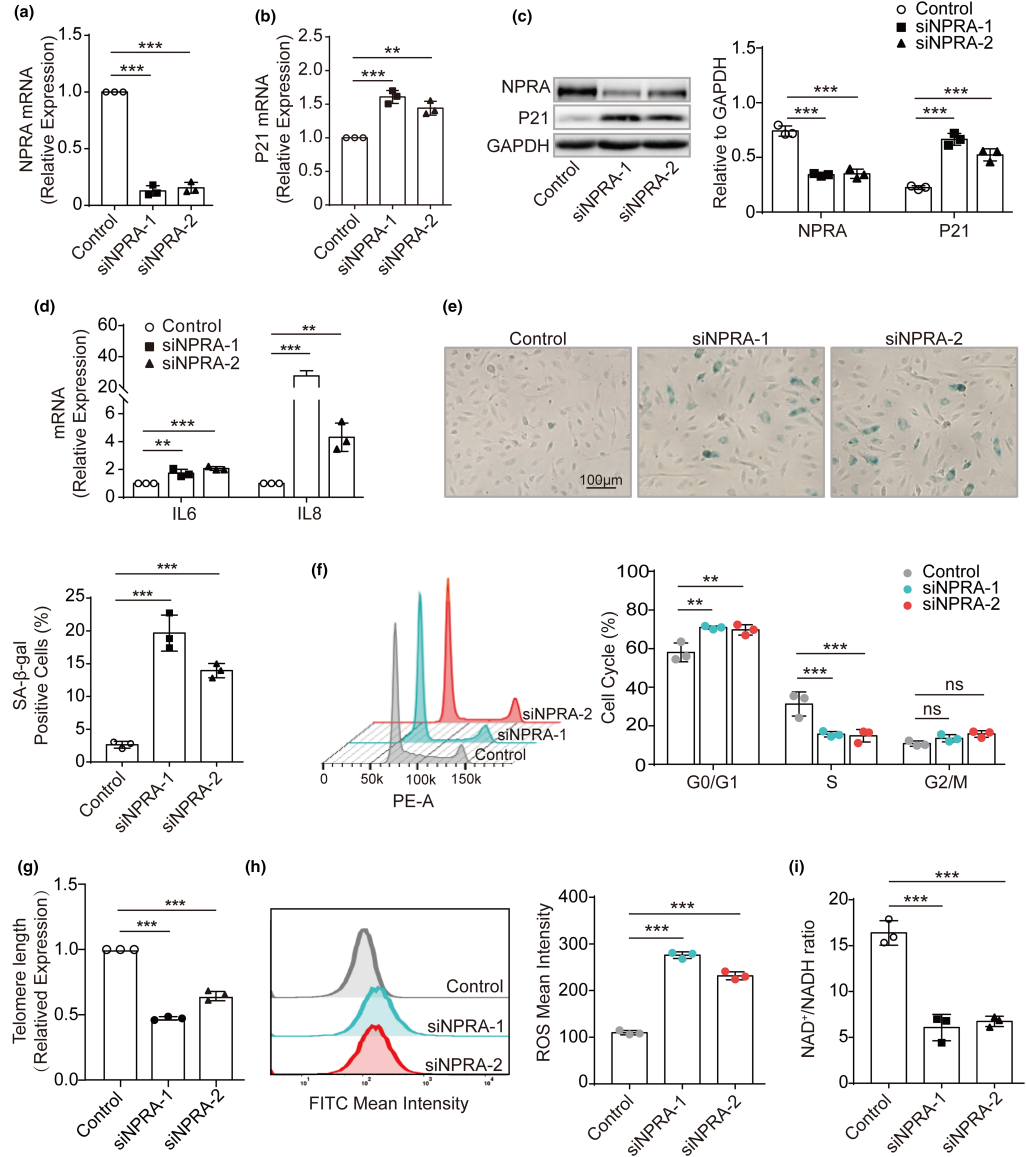
Increased endothelial cellular senescence by NPRA gene knockdown. (a) mRNA expression of NPRA in HUVECs treated with two sets of siRNA targeting the human *NPRA* (siNPRA‐1 and siNPRA‐2) by qPCR. Statistical analysis using one‐way ANOVA. (b) qPCR quantification of mRNA encoding P21 in HUVECs transfected with two sets of siNPRA. One‐way ANOVA used for statistical analysis. (c) Western blot analysis of NPRA and P21 expression in HUVECs treated with two sets of siNPRA. Analysis of endogenous NPRA and P21 levels, normalized to GAPDH levels, by densitometric analysis. Statistical comparison by one‐way ANOVA. (d) qPCR quantification of mRNA encoding IL6 and IL8 in HUVECs transfected with two sets of siNPRA, respectively. Two‐way ANOVA used for statistical analysis. (e) SA‐β‐gal staining in HUVECs transfected with two sets of siNPRA and quantitative data for SA‐β‐gal‐positive cells. Statistical analysis by one‐way ANOVA. (f) Cell cycle analysis of HUVECs transfected with two sets of siNPRA by flow cytometer. Comparison of means for percentage of cell cycle progression by two‐way ANOVA. (g) Telomere length of HUVECs transfected with two sets of siNPRA by qPCR. One‐way ANOVA for statistical analysis. (h) ROS production from HUVECs transfected with two sets of siNPRA by flow cytometer in the FITC channel. Statistical significance of FITC‐ROS mean intensity analyzed by one‐way ANOVA. (i) the ratio of NAD^+^/NADH in HUVECs transfected with two sets of siNPRA by colorimetric analysis. One‐way ANOVA for comparison of means. Values are mean ± SD. **p < 0.01; ***p < 0.001; ns, nonsignificant

To determine whether the deficiency of NPRA results in endothelial cellular senescence, the expression of *NPRA* was knocked down with two sets of siRNA (siNPRA1 and siNPRA2) in HUVECs. NPRA mRNA was significantly decreased by siRNAs compared with scrambler group (Figure 2a). Subsequently, senescence‐related markers, including P21, SA‐β‐gal activity, and senescence‐associated secretory phenotype (SASP), were examined and we found that in the cells with *NPRA‐*knockdown, both mRNA and protein of P21 were upregulated (Figure 2b,c) and the SASP components, such as IL6 and IL8, were increased, compared with the controls (Figure 2d). The higher percentage of cells showed positive for SA‐β‐gal staining in exposure to siNPRAs exhibited (Figure 2e).

Additionally, we examined how *NPRA* deficiency impacted on endothelial functions. The flow cytometry analyses were performed and we detected that the cell cycle was arrested at G0/G1 phase (Figure 2f) when cells were transfected with siNPRAs. Furthermore, the telomere attrition (Figure 2g), the increased ROS production (Figure 2h) and decreased the ratio of NAD^+^/NADH (Figure 2i) were observed in siNPRA‐treated HUVECs.

These demonstrates that the decreased expression of *NPRA* cause HUVECs premature cellular senescence and dysfunction.

### 2.3 PKG activator 8‐Br‐cGMP attenuates endothelial senescence induced by *NPRA* ‐knockdown

**FIGURE 3:**
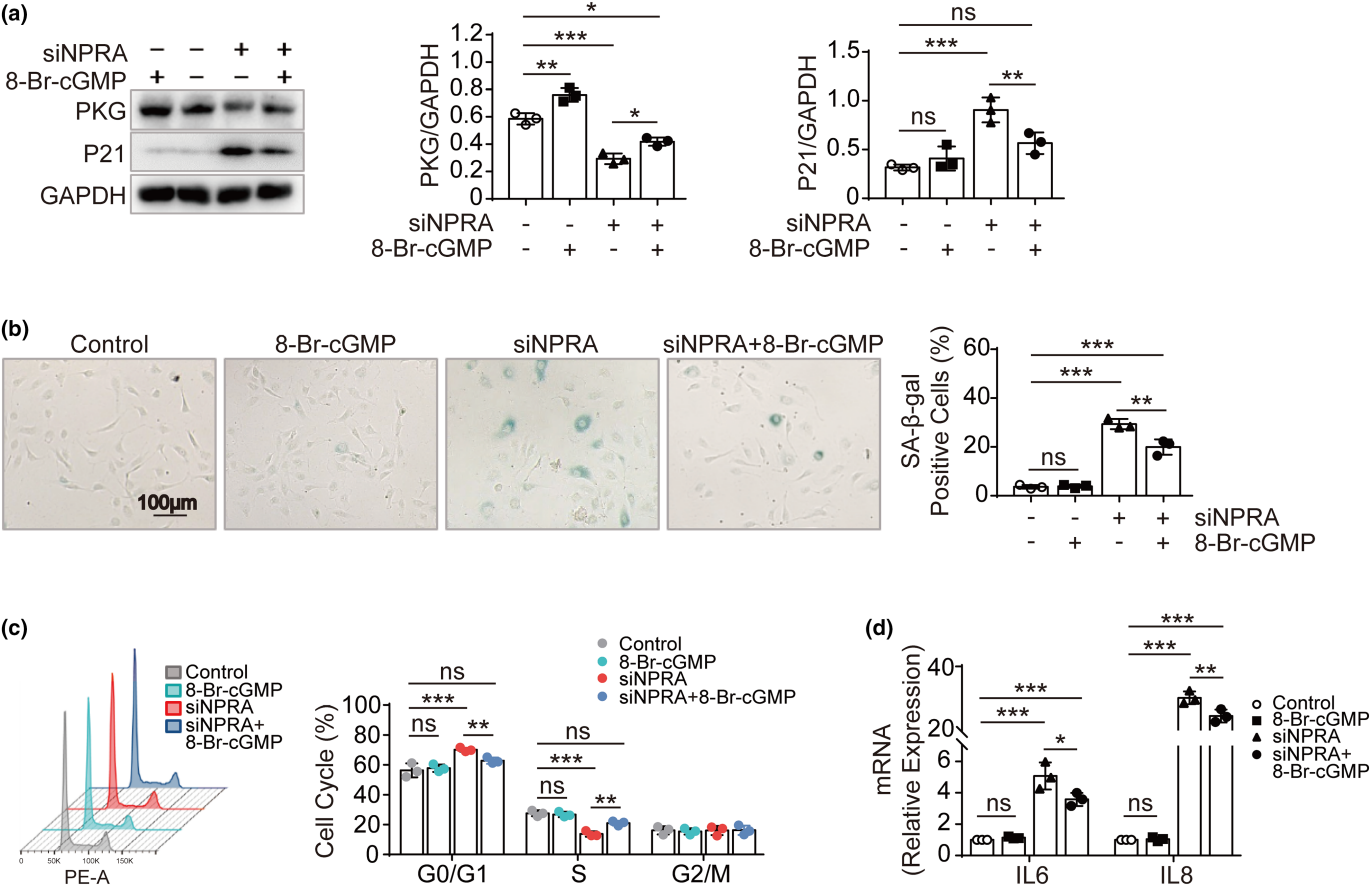
Rescue of *NPRA*‐knockdown induced endothelial senescence by PKG activator. (a) Western blot analysis of NPRA and P21 expression in HUVECs transfected with siNPRA and followed by treatment of PKG activator 8‐Br‐cGMP. Analysis of endogenous PKG and P21 level, normalized to GAPDH, by densitometry, respectively. One‐way ANOVA for comparison of means. (b) SA‐β‐gal staining in HUVECs transfected with siNPRA and then treated with 8‐Br‐cGMP. Percentage of SA‐β‐gal‐positive cells calculated. Comparison of means by one‐way ANOVA. (c) qPCR quantification of IL6 and IL8 in HUVECs transfected with siNPRA and then treated with 8‐Br‐cGMP, respectively. Two‐way ANOVA used for statistical analysis. (d) Cell cycle analysis of HUVECs transfected with siNPRA and then treated by 8‐Br‐cGMP. Statistical analysis for percentage of cell cycle progression by two‐way ANOVA. Values are mean ± SD. *p < 0.05; **p < 0.01; ***p < 0.001; ns, nonsignificant

PKG is a direct downstream molecule of NPRA in signaling transduction, while 8‐Br‐cGMP is a cGMP analog that activates PKG. To determine whether NPRA‐induced senescence is mediated by PKG, we treated HUVECs transfected with siNPRA or/and 8‐Br‐cGMP and found that PKG protein level was elevated and that P21 expression and SA‐β‐gal‐positive cells were reduced remarkably in the group treated with both siNPRA and 8‐Br‐cGMP than that in the siNPRA‐transfected group (Figure 3a,b). In addition, the cell cycles were recovered and the increased IL6 and IL8 were suppressed when treated with 8‐Br‐cGMP (Figure 3c,d).

These findings indicate that the activation of PKG prevents cellular senescence and reverses endothelial cell dysfunction induced by *NPRA*‐knockdown.

### 2.4 AMPK is critical to the development of NPRA/PKG‐induced senescence

**FIGURE 4:**
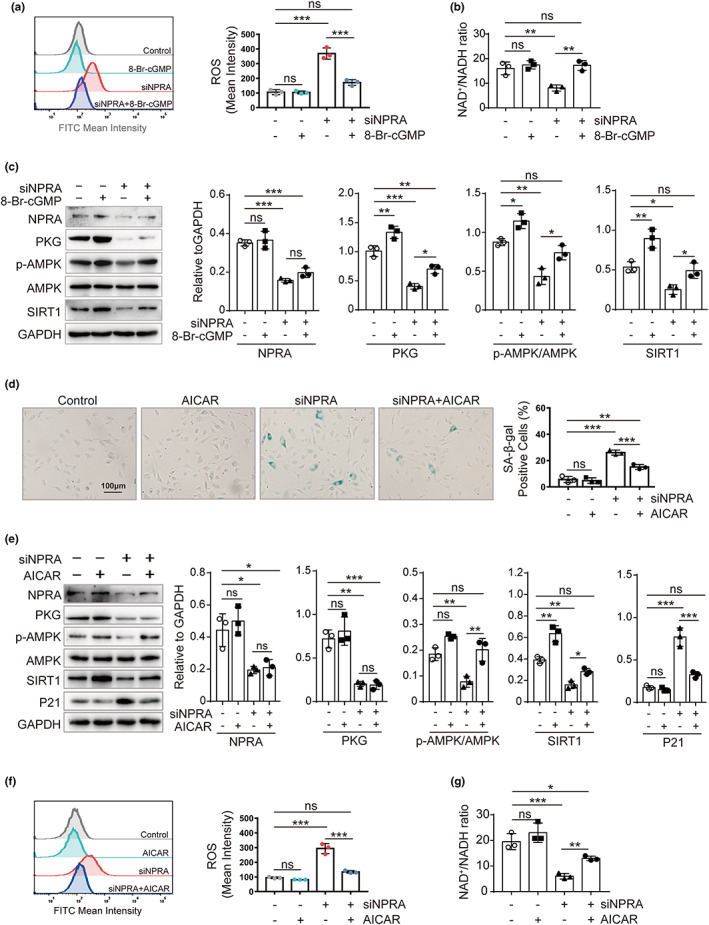
Impact of AMPK on the development of NPRA/PKG‐induced senescence. (a) ROS production in HUVECs treated with siNPRA and followed by treatment of 8‐Br‐cGMP as detected by flow cytometer in the FITC channel. Statistical analysis for FITC‐ROS mean intensity by one‐way ANOVA. (b) Colorimetric analysis for the ratio of NAD^+^/NADH in HUVECs transfected with siNPRA and followed by administration of 8‐Br‐cGMP. One‐way ANOVA for comparison of means. (c) Western blot analysis for the protein expression of NPRA, PKG, p‐AMPK, AMPK, and SIRT1 in HUVECs transfected with siNPRA and then treated by 8‐Br‐cGMP. Endogenous NPRA, PKG, p‐AMPK/AMPK ratio, and SIRT1, normalized to GAPDH, were analyzed by densitometry, respectively. One‐way ANOVA for comparison of means. (d) SA‐β‐gal staining in HUVECs transfected with siNPRA and then treated with AICAR. Percentage of SA‐β‐gal‐positive cells calculated. Comparison of means by one‐way ANOVA. (e) Western blot analysis for the protein expression of NPRA, PKG, p‐AMPK, AMPK, SIRT1, and P21 in HUVECs transfected with siNPRA and followed by treatment of AICAR. Endogenous NPRA, PKG, p‐AMPK/AMPK ratio, SIRT1, and P21, normalized to GAPDH, were analyzed by densitometry, respectively. One‐way ANOVA for comparison of means. (f) ROS production in HUVECs treated with siNPRA and followed by treatment of AICAR as detected by flow cytometer in the FITC channel. Statistical analysis for FITC‐ROS mean intensity by one‐way ANOVA. (g) Colorimetric analysis for the ratio of NAD^+^/NADH in HUVECs transfected with siNPRA and followed by administration of AICAR. One‐way ANOVA for comparison of means. Values are mean ± SD. *p < 0.05; **p < 0.01; ***p < 0.001; ns, nonsignificant

As observed that AMPK‐related components, including ROS production and the ratio of NAD^+^/NADH, were changed by 8‐Br‐cGMP treatment (Figure 4a,b), we tested if AMPK mediates the downstream signaling of NPRA/PKG in senescence. First, the Western blot showed that PKG and SIRT1 as well as p‐AMPK/AMPK were decreased by siNPRA and 8‐Br‐cGMP reversed these changes (Figure 4c). Next, AICAR, an activator of AMPK, was used to test its ability to rescue endothelial cell senescence promoted by *NPRA‐*knockdown. It was found AICAR significantly reduced the numbers of SA‐β‐gal stained cell and the expression of P21 induced by siNPRA and it increased p‐AMPK/AMPK and SIRT1 in siNPRA‐treated HUVECs (Figure 4d,e). Further, AICAR reduced ROS production and increased the ratio of NAD^+^/NADH (Figure 4f,g). Thus, our results support that NPRA regulates endothelial cell senescence through PKG‐AMPK‐SIRT1 signaling cascade.

### 2.5 *Npr1* ^+/−^ mice presents vascular aging that is reversed by activation of PKG

**FIGURE 5:**
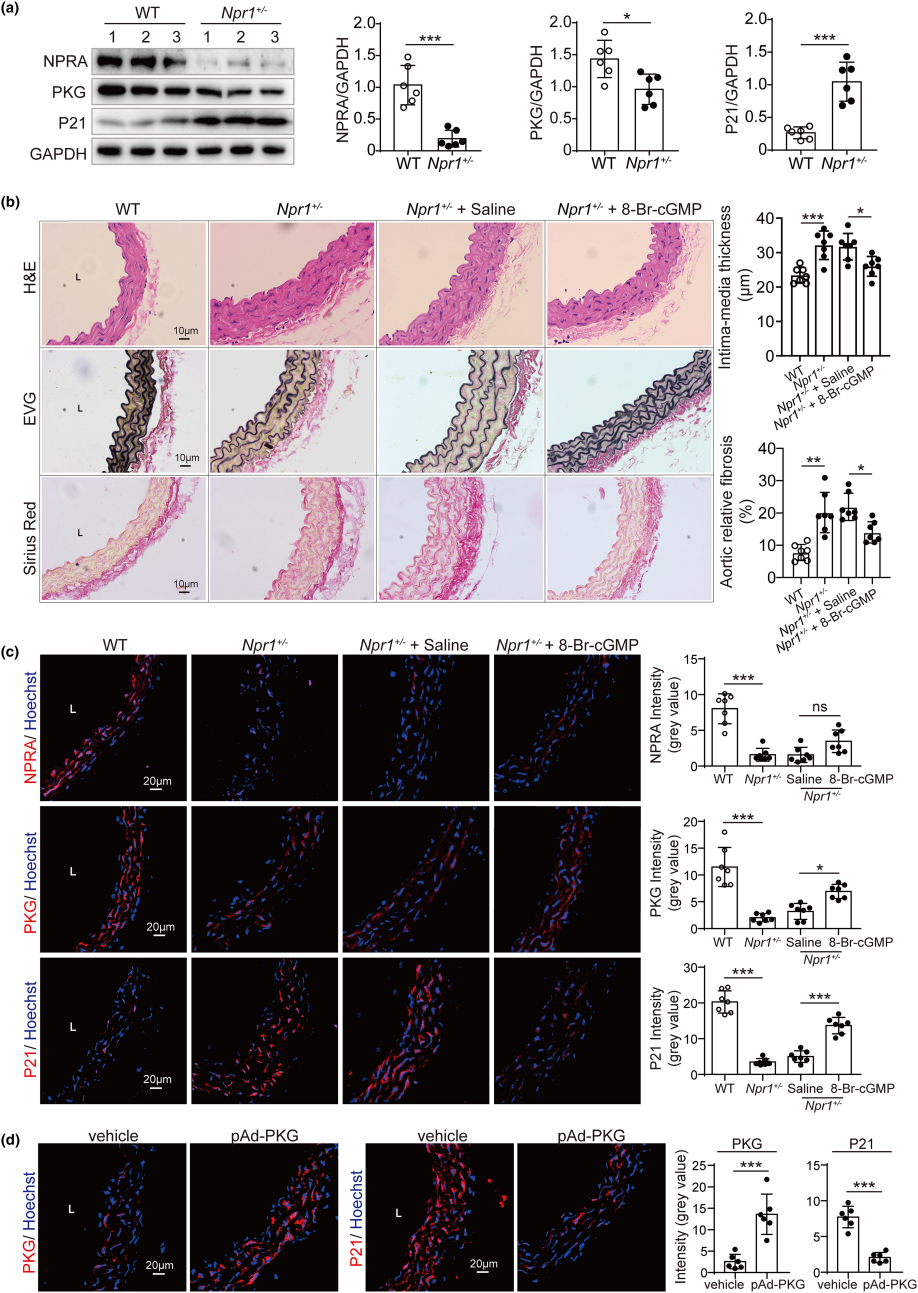
Reversal of vascular aging in *Npr1* ^*+/−*^ mice by activation of PKG. (a) Western blot analysis of NPRA, PKG, and P21 expression in the aorta from WT, *Npr1* ^+/−^ mice (*n* = 7, at 7–8 months of age). Endogenous NPRA, PKG, and P21 levels, normalized to GAPDH levels, by densitometric analysis. Statistical comparison by Student's *t*‐test. (b) Histological features of the aorta from WT, *Npr1* ^+/−^, and *Npr1* ^+/−^ treated with saline or 8‐Br‐cGMP mice (*n* = 7, at 7–8 months of age) by staining of H&E for structure, EVG (elastic Van Gieson) for elastic fibers (black), and Sirius red for collagen (sirius red). Statistical comparison by one‐way ANOVA. (c) Immunofluorescence staining of the aorta from WT, *Npr1* ^+/−^, and *Npr1* ^+/−^ treated with saline or 8‐Br‐cGMP mice (*n* = 7, at 7–8 months of age) by antibodies against NPRA, PKG and P21 (red). Nuclei (blue) stained by Hoechst. The mean intensity was measured, and statistical comparison was performed by one‐way ANOVA. (d) Immunofluorescence staining of the aorta from WT, *Npr1* ^+/−^, and *Npr1* ^+/−^ treated with saline or pAd‐PKG mice (*n* = 7, at 7–8 months of age) by antibodies against PKG and P21 (red). Nuclei (blue) stained by Hoechst. The mean intensity was measured, and statistical comparison was performed by Student's *t‐*test. Values are mean ± SD. *p < 0.05; **p < 0.01; ***p < 0.001; ns, nonsignificant.

Our *in vitro* study revealed that PKG plays a role in endothelial cellular senescence associated with NPRA. We attempted to replicate this finding in vivo. For this purpose, we developed *Npr1* knockout mouse model with CRISP/CAS9 technique and observed no gross defects in adult *Npr1* ^+/−^ mice. However, *Npr1* ^−/−^ mice died during postnatal stage, restricting this study to *Npr1* ^+/−^ mice. By Western blot analysis, it was found that P21 was increased in *Npr1* ^+/−^ mice comparing with WT mice at the age of 7–8 months (Figure 5a). Next, morphological changes of the aorta tissues were compared in H&E, EVG, and Sirius red staining in *Npr1* ^+/−^ with littermate control treated with saline or 8‐Br‐cGMP. It was found that the aorta of *Npr1* ^+/−^ mice had a thickened wall, thinner and disrupted elastic fibers, and apparent collagen accumulation, while these aging phenotypes were reversed after *Npr1* ^+/−^ mice were treated by 8‐Br‐cGMP that stimulated PKG activity (Figure 5b), suggesting that decrease of NPRA‐induced vascular aging through PKG.

Finally, human PKG gene was overexpressed by adenoviral infection in *Npr1* ^+/−^ mice. Similar to that observed with 8‐Br‐cGMP, the adenoviral expressions of PKG reduced P21 expression significantly (Figure 5c,d).

These results suggest that deficiency of NPRA induces but the activation of PKG reverses vascular aging in vivo.

### 2.6 Activation of PKG improves endothelium‐dependent vascular relaxation impaired in *Npr1* ^+/−^ mice

**FIGURE 6:**
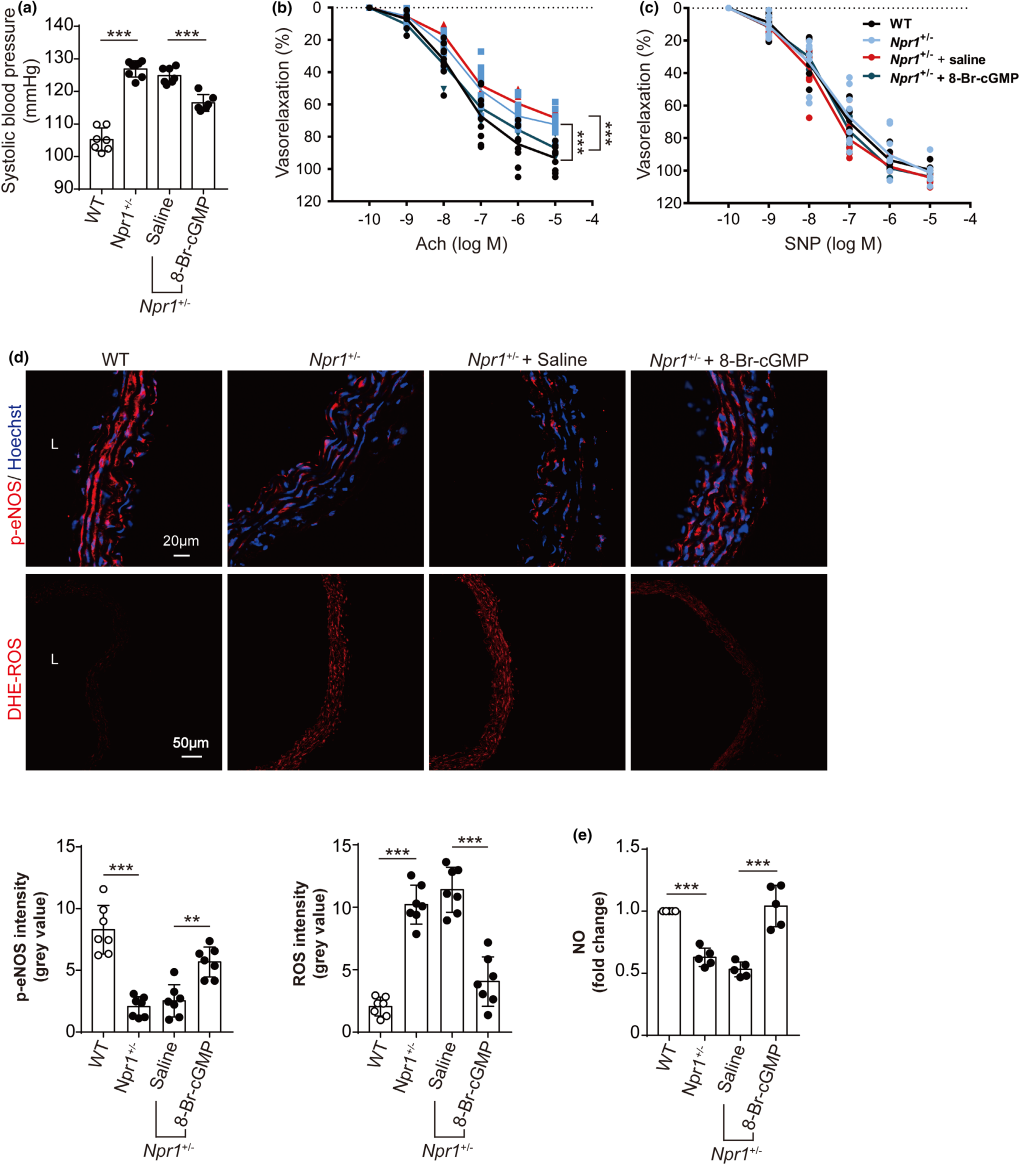
Activation of PKG in the improvement of endothelium‐dependent vascular relaxation and decrease of blood pressure in *Npr1* ^+/−^ mice. (a) Systolic blood pressure of WT (*n* = 7), *Npr1* ^+/−^ (*n* = 8), and *Npr1* ^+/−^ treated with saline (*n* = 7) or 8‐Br‐cGMP (*n* = 6) mice at the age of 7–8 months. One‐way ANOVA for statistical comparison. (b) Endothelium‐dependent vascular relaxation induced by acetylcholine (Ach) for the aorta rings from WT, *Npr1* ^+/−^, and *Npr1* ^+/−^ treated with saline or 8‐Br‐cGMP mice (*n* = 6) at the age of 7–8 months. The cumulative concentration response curves were recorded, and statistical significance was analyzed by one‐way ANOVA. (c) Endothelium‐independent vasorelaxation mediated by nitroprusside (SNP) for the aorta rings from WT, *Npr1* ^+/−^, and *Npr1* ^+/−^ treated with saline or 8‐Br‐cGMP mice (*n* = 6) at the age of 7–8 months. The cumulative concentration response curves were recorded, and statistical significance was analyzed by one‐way ANOVA. (d) Detection of phospho‐eNOS (p‐eNOS) and ROS production in the aorta from WT, *Npr1* ^+/−^, and *Npr1* ^+/−^ treated with saline or 8‐Br‐cGMP mice (*n* = 7) at the age of 7–8 months using antibody against p‐eNOS and ROS fluorescent probe‐DHE. Nuclei (blue) stained by Hoechst. Statistical comparison for the mean intensity by one‐way ANOVA. (e) Colorimetric analysis for NO for the aorta from WT, *Npr1* ^+/−^, and *Npr1* ^+/−^ treated with saline or 8‐Br‐cGMP mice (*n* = 5) at the age of 7–8 months. One‐way ANOVA for comparison of means. Values are mean ± SD. **p < 0.01; ***p < 0.001

To evaluate whether 8‐Br‐cGMP rescues vascular functional defect in *Npr1* ^+/−^ mice, we measured the blood pressure and vascular contractile functions. It was observed that *Npr1* ^+/−^ mice had an elevated systolic blood pressure that was able to be lessened by 8Br‐cGMP (Figure 6a). The vasodilation of aorta rings in response to Ach in *Npr1* ^+/−^ mice was significantly declined and was reverted by 8‐Br‐cGMP (Figure 6b), whereas the vasodilation of aorta rings from WT, *Npr1* ^+/−^, and *Npr1* ^+/−^ plus 8‐Br‐cGMP mice showed similar response to SNP (Figure 6c). Further, we showed that the decreased p‐eNOS and NO as well as increased ROS in the aorta from *Npr1* ^+/−^ mice were normalized by the administration of 8‐Br‐cGMP (Figure 6d,e).

These findings suggest that a prohibition of NPR1 impairs endothelium‐dependent vascular relaxation and decreases the production of NO in *Npr1* ^+/−^ mouse model.

### 2.7 Stimulation of NPRA/PKG reverses endothelial cell senescence and vascular aging

**FIGURE 7:**
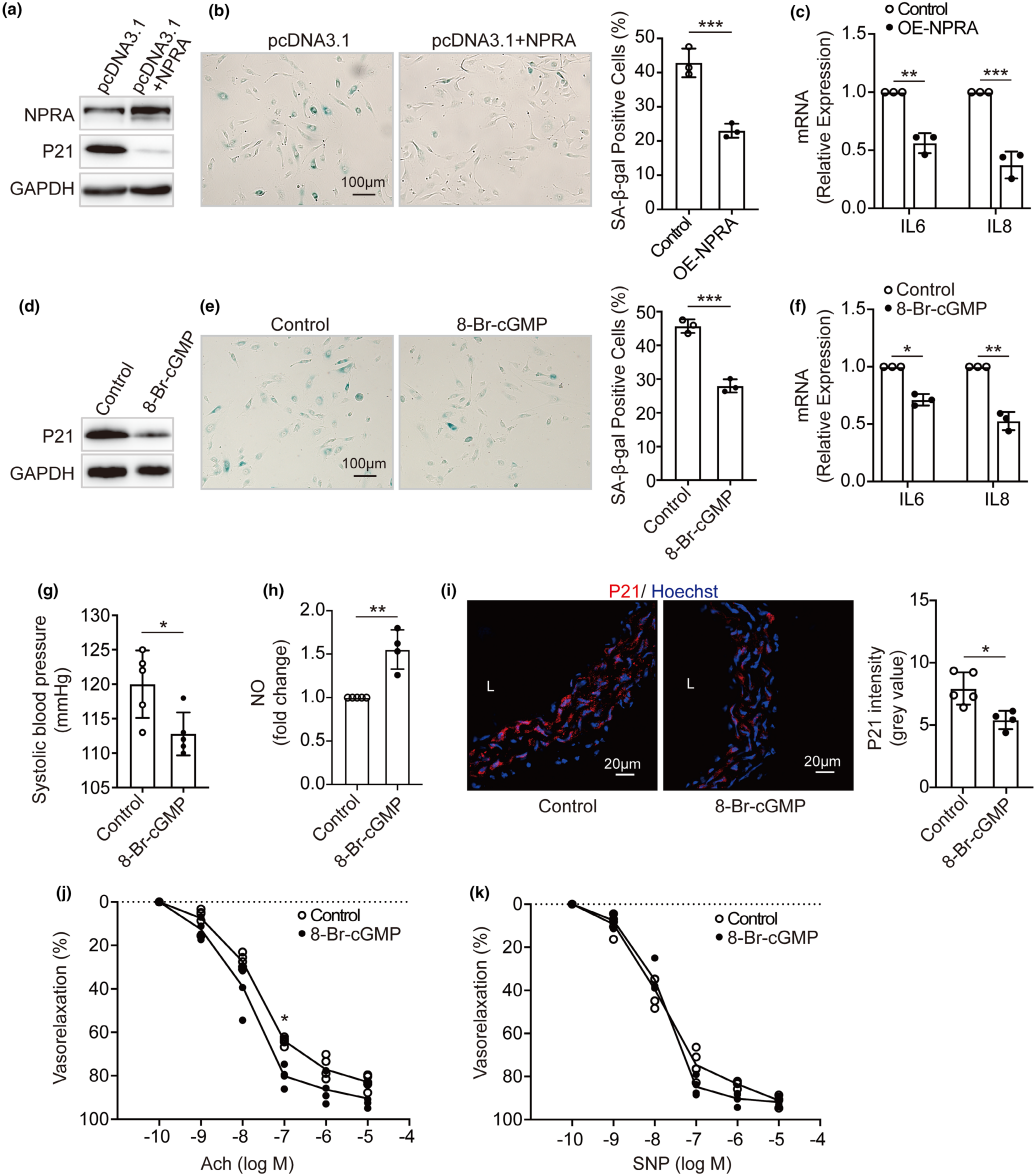
Reversal of endothelial cell senescence and vascular aging by activating NPRA‐PKG. (a) Western blot analysis of NPRA and P21 expression in the senescent HUVECs (PDL 38) transfected with a plasmid encoding human NPRA. (b) SA‐β‐gal staining in NPRA‐overexpressed HUVECs (PDL 38). Percentage of SA‐β‐gal‐positive cells estimated and comparison of means by Student's *t*‐test. (c) IL6 and IL8 levels in NPRA‐overexpressed HUVECs (PDL 38). Student's *t*‐test used for statistical analysis. (d) Protein expression of P21, (e) SA‐β‐gal activity, and (f) IL6 and IL8 levels in the senescent HUVECs (PDL 38) treated by 8‐Br‐cGMP. Student's *t*‐test for comparison of means. (g) Systolic blood pressure of WT mice (*n* = 5) treated with 8‐Br‐cGMP or saline as a control at the age of 13–15 months. Student's *t*‐test for statistical comparison. (h) NO levels for the aorta from WT mice (13–15 months) treated with saline (*n* = 5) or 8‐Br‐cGMP (*n* = 4). Student's *t*‐test for comparison of means. (i) Immunofluorescence staining for P21 (red) and nuclei (blue) with frozen aorta tissue sections from WT mice (13–15 months) treated with saline (*n* = 5) or 8‐Br‐cGMP (*n* = 4). Statistical comparison by Student's *t*‐test. (j) Endothelium‐dependent vasorelaxation induced by acetylcholine (Ach) and (k) Endothelium‐independent vasorelaxation mediated by nitroprusside (SNP) for the aorta rings from WT mice (13–15 months) treated with saline (*n* = 5) or 8‐Br‐cGMP (*n* = 4). The cumulative concentration response curves were recorded, and statistical analysis was executed using one‐way ANOVA. Values are mean ± SD. *p < 0.05; **p < 0.01; ***p < 0.001

To validate the effect of NPRA/PKG on vascular endothelial senescence, we performed the experiments in the senescent HUVECs (PDL 38) and in aged mouse model (13–15 months). We observed that the senescent cells exposed to a plasmid encoding human NPRA exhibited a significant reduction in the expression of genes, including P21, IL6, and IL8 as well as in the number of cells stained with SA‐β‐gal, compared with those transfected with a control plasmid only (Figure 7a–c). Similar results were obtained from the senescent cells treated by 8‐Br‐cGMP (Figure 7d–f). Moreover, we found that high systolic blood pressure in aged mice was considerably decreased after treatment of 8‐Br‐cGMP (Figure 7g). The reduction of NO and increased P21 in the aorta of aged mice were reverted in the presence of 8‐Br‐cGMP (Figure 7h,i). The declined vascular relaxation in response to Ach in aged aorta was improved by 8‐Br‐cGMP (Figure 7j). However, there is no difference in vasodilation in response to SNP between control and 8‐Br‐cGMP mice (Figure 7k). These data suggests that overexpression of NPRA and treatment of 8‐Br‐cGMP rescue senescence in HUVECs and mouse model, demonstrating that NPRA/PKG signaling axis may prevent endothelial senescence and vascular aging.

## 3 DISCUSSION

We, in this study, show that the expression of *NPRA* gene is chronologically decreased and this decrease promotes endothelial cell senescence, vascular aging, and hypertension through NPRA/PKG/AMPK signaling axis.

Existing evidence supports that aging is a strong and independent risk factor for cardiovascular diseases (Tian & Li, 2014), including hypertension, the most common cardiovascular disease in elderly population worldwide (McCarthy et al., 2019). Hypertension has been considered as a phenotype of premature vascular aging (Schreckenberger et al., 2020). Senescence likely contributes to the development of hypertension as the senescent cells are present in hypertensive vessels (Oeseburg et al., 2009; Sueta et al., 2014). Plasma ANP is positively increased with ages in man and rat models (Korytkowski & Ladenson, 1991; Tonolo et al., 1989). It is, however, observed that the natriuretic or hypotensive response to exogenous ANP become decreased in elderly man and aged rodents (Lai et al., 2000; Mulkerrin et al., 1993; Pollack et al., 1997). These indicate a defect in ANP‐initiated signaling pathway during aging. Here, we show that the receptor of ANP (NPRA) is down‐regulated during chronological aging, thus the ANP‐mediated signaling cannot be fully conducted in senescent cell and aged vessel. We further demonstrate that the decreased NPRA promotes endothelial cell senescence, vascular aging, and blood pressure elevation, providing evidence that these is an intrinsic link among senescence, aging, and hypertension.

This study was initially designed to search for genes that contribute to chronological vascular aging and hypertension. For this purpose, RNA‐seq on two batches of HUVECs during replicative senescence was performed and as a consequence, 25 genes were found to be changed in their transcripts during senescence and were enriched under the term of “regulation of blood pressure” in GO analyses, becoming candidate genes for further studies. Thus, instead of screening all candidates for their causative relationship with endothelial cell senescence, we focused on *NPRA* gene as it was abundant in endothelial cell and decreased most significantly. It has been well‐documented that null‐function of *NPRA* in the mouse model not only elevates blood pressure but also increases atherosclerosis and inflammation, two phenotypes that are tightly linked to vascular aging (Merat et al., 2000; Minamino et al., 2002; Oliver et al., 1997). In fact, the published GEO DataSets from microarray (GSE45541)‐ or RNA sequencing (GSE130727, GSE163251, and GSE17506) reinforced the finding that NPRA is decreased during endothelial cell senescence (Casella et al., 2019; Wong et al., 2017; Zhang et al., 2021). Another reason why we prioritized NPRA for further study was that we have previously showed that ANP‐processing enzyme CORIN decreases blood pressure and that ANP is the ligand of NPRA (Chen et al., 2015). In addition, we demonstrated here that NPRA was decreased in aged aorta, providing an *in vivo* evidence to bridge the missing link. These together strongly support that NPRA is a plausible candidate that links vascular aging and hypertension.

Whether or not NPRA contributes to cellular senescence has not been previously reported. In early studies, it has been shown that decreased NPRA expression prohibited proliferation of vascular smooth muscle cell (Gao et al., 2009), suggestive of the critical role of NPRA pathway in regulation of the cell cycle. Here, we demonstrated that decreased NPRA promoted endothelial cell senescence, as cells with decreased NPRA presented typical features observed in senescence (Tian & Li, 2014), including an arrested cell cycle, increased SA‐β‐gal activity and expression of *P21*, promoted SASP (IL6 and IL8), telomere shortening, and cellular dysfunction (increased ROS but decreased NAD^+^/NADH ratio; Guo et al., 2017; Hayashi et al., 2006; Silva et al., 2017). We further observed that vessels from mice heterozygous knockout for *NPRA* exhibited an increased expression of *P21* and collagen, disorganized elastin (thinner and broken), thickened Intima‐media layer, desensitized to acetylcholine‐mediated vasodilation, increased ROS production, decreased expression of *eNOS* and *SIRT1* as well as NO release, in addition to the elevated blood pressure. These observations are frequently reported in vessels with advanced age in man and model organisms, suggesting that mice heterozygous for *NPRA* present age‐related vascular phenotypes (Bruno et al., 2017; Gerhard‐Herman et al., 2011; Panza et al., 1990; Soltis, 1987; Staudt et al., 2020; Yang et al., 2020). Importantly, these phenotypic changes were rescued by overexpressed NPRA and 8‐Br‐cGMP (an analog of cGMP; Rodriguez‐Pascual et al., 1994) and overexpression of PKG (Airhart et al., 2003), providing solid evidence that NPRA/cGMP/PKG, a well‐documented signaling pathway for blood pressure regulation (Oliver et al., 1997, 1998; Pandey, 2018), has a novel function in the modulation of endothelial cell senescence and vascular aging. It should be mentioned that this study focused on mice heterozygous for NPRA as homozygous null‐function of *NPRA* led to postnatal death (Oliver et al., 1997).

Another important finding in this study is that AMPK was defined as downstream node molecule for NPRA‐cGMP‐PKG pathway in the control of endothelial cell senescence, vascular aging, and blood pressure. First, the phosphorylation of AMPK was decreased in endothelial cell with knockdown of *NPRA* while 8‐Br‐cGMP treatment enhanced the phosphorylation. Second, the transcripts of *SIRT1*, *eNOS*, and *P21*, as well as the level of NAD^+^ and ROS, which are AMPK‐associated components, were accordingly changed in response to NPRA and PKG both *in vitro* and *in vivo*. Finally, the activation of AMPK with AICAR prevented endothelial cell senescence caused by decreased NPRA. These suggest that NPRA/PKG/AMPK signaling axis is novel but critical to control cellular senescence and vascular aging.

AMPK, an AMP‐activated protein kinase, serves as a nutrient and energy sensor critical to the maintenance of energy homeostasis (Hardie et al., 2012). That AMPK contributes to senescence and aging has been reported through multiple pathways or feedback loops, including energy control (Karnewar et al., 2018), autophagy (Zhang et al., 2019), and protein synthesis (van Vliet et al., 2021; Zhan et al., 2018), and eventually act on regulators of cell cycle (Baek et al., 2020; McKay & White, 2021). In replicative senescence presented in this study, AMPK regulates senescence most likely through energy control as NAD^+^, SIRT1, and ROS were found to be involved and particularly the activation of the Sirt1‐AMPK‐eNOS has been reported to slow down vascular stiffness (Chen & Sun, 2018; Han et al., 2016; Wiley & Campisi, 2016). Although it has been known that kinases, such as LKB1, CAMKK2, and ATM, phosphorylate and activate AMPK, there are no reports that PKG phosphorylates AMPK directly. Interestingly, the published studies showed that NPRA‐PKG signaling cascade promoted muscle mitochondrial biogenesis, lipid metabolism, and oxygen utilization through AMPK, demonstrating an existing link from natriuretic peptide to AMPK pathway (Benitez‐Amaro et al., 2020; Ding et al., 2019; Li et al., 2016; Souza et al., 2011). AMPK and its components form very complicated signaling loops or networks (Harada et al., 2012; Mihaylova & Shaw, 2011; Wiley & Campisi, 2016); therefore, the consequence of activating AMPK in delaying senescence or aging should be the net effect. We show here that activation of AMPK prevents the decreased NPRA‐induced senescence and vascular aging, demonstrating it as a central downstream molecule of NPRA. As AMPK is involved in the regulation of blood pressure (Chen & Sun, 2018), NPRA/PKG/AMPK possibly plays dual functions in blood pressure control and vascular aging.

This study has several limitations. First, we searched for genes that are shifted during replicative HUVEC senescence. HUVECs is the commonly used model for studying endothelial functions, including senescence. However, it may not fully represent endothelial cell from a small artery. Second, our study focused on NPRA, which is changed most significantly in multiple omics datasets. This may ignore other important genes that contribute to both vascular aging and hypertension. Finally, the contribution of NPRA/PKG/AMPK pathway to human vascular aging and hypertension has yet to be evaluated.

In summary, we report that NPRA/PKG/AMPK is a novel but critical signaling axis in control of cellular senescence and vascular aging. As NPRA/PKG/AMPK signaling is chronologically prohibited, our finding provides a plausible explanation how aging contributes to age‐related diseases, indicating that this pathway may have implications in prevention of age‐related vascular diseases, such as hypertension, by slowing down aging process.

## 4 EXPERIMENTAL PROCEDURES

### 4.1 Cell culture

HUVECs were isolated from fresh umbilical cord of various donors and cultured in Endothelial Cell Medium (ECM; Hyclone) according to our previous report (Li et al., 2018). This research was approved by the Ethics Committee of Nanchang University (HARI‐SC‐0061) and written informed consent was obtained from the fathers of donors.

### 4.2 RNA sequencing

Total RNA was isolated from the cultured HUVECs from two donors A (Batch_A) and B (Batch_B) at population doubling levels 12 (PDL 12, young) and 39 (PDL 39, senescent) using TRIZOL reagent method (Invitrogen), respectively. Library was made with Illumina Truseq RNA Sample Preparation v2 Kit, then sequencing was carried out with Illumina HiSeq 2000 platform at BIOPIC at Peking University.

Sequencing reads that contained adapters or had low quality were pre‐filtered before mapping. Filtered reads were mapped to the hg19 genome using Tophat2 software (Version 2.0.11; Kim et al., 2013). Relative expression levels (reads per kilobase per million mapped reads, RPKM) were calculated by Cufflinks software (Version 2.2.1; Trapnell et al., 2010). Genes with RPKM <2 at both PDL12 and 39 were excluded in further analyses. The gene is defined as “transcriptionally changed or shifted” as its transcript is changed twofold in both Batch_A and Batch_B.

The senescence‐altered genes were subjected to enrichment analyses at Gene Ontology (GO; http://david.abcc.ncifcrf.gov/; Huang da et al., 2009).

### 4.3 Quantitative real‐time PCR (qPCR)

Total RNAs were extracted from cultured cells and mouse tissues with TRIzol reagent (Bmassay) and cDNA was synthesized using a reverse transcriptase kit (Zomanbio). The expression of genes, including *NPRA*, interleukin 6 and 8 (*IL6* and *IL8*), and *CDKN1A* (*P21*), and telomere length were detected and quantified by Realtime PCR Super mix‐SYBR Green with anti‐Taq kit (Mei5bio). The data were normalized to β‐actin gene. The primer sequences were provided in Supporting Information (Table S1).

### 4.4 Western blot

Cell or tissue was lysed using RIPA lysis buffer (Bmassay), and Western blot was carried out based on published literature (Li et al., 2019). In brief, protein extracts were separated in 10% SDS polyacrylamide gel and transferred onto polyvinylidene fluoride (PVDF) membranes (Millipore). After blocking the membrane for 1 h, primary antibody was incubated overnight with gentle agitation. The primary antibodies for the target proteins included NPRA (Fabgennix), P21 (Proteintech), sirtuin 1 (SIRT1), PKG, AMPK and phosphorylated‐AMPK (p‐AMPK; Cell Signaling Technology). Subsequently, secondary antibody goat anti‐rabbit IgG or goat anti‐mouse IgG (ABclonal) was incubated at room temperature for 1 h. The blots were visualized employing a BeyoECL Moon kit (Beyotime). For probing multiple targets with the same membrane, stripping and re‐probing were performed. Briefly, the PVDF membrane was incubated with the stripping buffer containing 62.5 mM Tris–HCl (pH 6.8), 2% SDS and 100 mM β‐mercaptoethanol for 20 min at room temperature. After washing three times with TBST, the membrane was re‐incubated with primary and secondary antibodies.

The protein levels, normalized with GAPDH (Proteintech), were calculated by ImageJ (NIH).

### 4.5 Senescence‐associated‐β‐galactosidase (SA‐β‐gal) staining

SA‐β‐gal staining was conducted using a Senescence Detection Kit (Beyotime). Cells were harvested and washed with PBS, incubated in a fixative solution for 15 min, and then stained with in SA‐β‐gal reaction solution at 37°C for 16 h. The blue‐stained cells were identified as senescent cells and the number of the senescent cells were counted under microscope in fifteen fields.

### 4.6 RNA interference and plasmid transfection

When reaching to 60%–70% confluency, 50 nmol/L of siRNA oligonucleotides or 0.5 μg/ml of pcDNA 3.1 plasmid encoding human NPRA were transfected into HUVECs with Lipofectamine 2000 (Invitrogen) in Opti‐MEM (Gibco), according to the manufacturer's instructions. The sequences of targeting siRNA against human *NPRA* gene as follows: siNPRA‐1: 5′‐GCAAAGGCCGAGUUAUCUA‐3′; siNPRA‐2: 5′‐CCUAUGAGCAG UUCAACUU‐3′. The sequences of non‐target scramble controls were provided by Genepharma. At 72 h after transfection, the cells were collected for further experiments.

### 4.7 Cell cycle analysis

At 32 h after transfection, the cells were separated by trypsinization. After three washes in PBS, the cells were fixed with 70% ethanol at 4°C overnight. Then, they were stained with 75 μmol/L propidium iodide (Sigma) and 3.6 μmol/L RNase at 37°C for 30 min. The distribution of cell cycles was detected by FACSVerse flow cytometer (BD Bioscience) and the proportion was analyzed using flowjo v10 software (BD Bioscience).

### 4.8 Mouse models

Mice with C57BL/6 background were used to generate *Npr1‐*knockout model using CRISPR/Cas9 technique (Bioray Laboratories Inc). *Npr1* is an orthologous gene of human *NPRA*. *Npr1* ^+/−^ mice from 7 to 8 months of age and their littermates were used for blood pressure measurement, isometric tension measurement of thoracic aorta and tissue collection. Only male mice were used in this study. All animal procedures were approved by the Institutional Animal Care and Use Committee of Nanchang University (protocol No. HARI‐SC‐0067).

### 4.9 Blood pressure measurement

Systolic blood pressure in mice was measured using a non‐invasive tail‐cuff device IITC (Life science). Mice were trained with unrecorded measurement for one week to be adapted to the tail‐cuff process. The blood pressure was then taken every afternoon for three consecutive days. The measurement was performed in 10–15 s for each inflation and deflation cycle and 10 consecutive pulse readings were documented for each mouse.

### 4.10 Isometric tension measurement

Mouse thoracic aortas from mice were taken and perivascular adipose tissue were meticulously removed in modified Krebs–Henseleit solution (118 mmol/L NaCI, 4.7 mmol/L KCI, 2.5 mmol/L CaCl_2_, 1.2 mmol/L MgSO_4_, 1.2 mmol/L KH_2_PO_4_, 25 mmol/L NaHCO_3_, and 11 mmol/L glucose) at 37°C gassed with 95% O_2_ and 5% CO_2_. The aorta was cut into rings of 2–3 mm in length. The tension measurement was conducted using Myograph Pressure System DMT620M (Danish Myo Technology). The aorta rings were placed in an aerated bathing tube and phenylephrine (1.0 × 10^−6^ mol/L) was added to establish an initial vascular activity. When vascular activity was stabilized, the rings were equilibrated for 90 min at a constant basal tension of 3 mN. After the rings achieved maximum contraction, 10^−9^–10^−5^ mol/L of acetylcholine (Ach) or sodium nitroprusside (SNP) was administered to induce endothelium‐dependent relaxation or endothelium‐independent relaxation. The Ach‐ or SNP‐induced vasodilatation curves with cumulative concentrations were recorded and analyzed.

### 4.11 Administration of 8‐Br‐cGMP, AICAR, and pAd‐PKG

8‐Br‐cGMP (Sigma‐Aldrich) is cell‐permeable analog of cGMP. AICAR (SigmaAldrich) is an activator for AMPK. After HUVECs were incubated with siNPRA for 24 h, the cells were treated by 0.1 mmol/L of 8‐Br‐cGMP or 0.5 mmo/L of AICAR, respectively and subsequently cultured for 48–72 h. All the cells were examined by Western blotting and functional analysis.

In the animal study, aged mice (14 months) or *Npr1* ^+/−^ mice (6 months) weighing about 35 g were randomly divided into two groups. One group was administered with 0.1 mmol/L of 8‐Br‐cGMP that was diluted in 0.2 ml of normal saline through the tail vein. The other group was injected with 0.2 ml of normal saline as controls. Similarly, recombinant adenovirus vector expressing human PKG (pAd‐PKG; WZ Biosciences Inc.) was administered with 1 × 10^10^ PFU that was diluted in 0.2 ml of normal saline and pAd‐vector in normal saline was used as controls for tail vein injection for each group, respectively. All the treatments were performed every two weeks. A total of 4 injections were applied. One week after the fourth injection, the mice were sacrificed, and the thoracic aortas were isolated for the further experiments.

### 4.12 NAD ^+^/NADH measurement

Nicotinamide adenine dinucleotide (NAD) is a coenzyme including NAD^+^ (oxidized) and NADH (reduced) forms found in all cells. The NAD^+^/NADH ratio was examined using a commercial kit (Beyotime) according to the manufacturer's manual. Briefly, cells were lysed to determine total amount of NAD^+^ and NADH. Then, the NAD^+^ in the sample was decomposed at 60°C for 30 min to detect NADH content. In addition, NAD^+^ was quantified. The samples were loaded into a 96‐well plate for absorbance measurement at 450 nm. The ratio of NAD^+^/NADH was calculated.

### 4.13 Histochemistry and immunofluorescence

The thoracic aorta tissues were fixed with 4% paraformaldehyde for 24 h and then embedded in paraffin. The aorta sections were prepared with the descending aorta (after the origin of the left subclavian artery from the aortic arch) in 5 μm thickness and subsequently stained with H&E for structure, EVG (Elastic Van Gieson) for elastic fibers and Sirius Red for collagen deposition. The images were taken under a light microscope.

The thickness of intima‐media and degree of fibrosis were analyzed by Image J.

For immunofluorescent analysis, thoracic aorta tissues were embedded in Tissue Freezing Medium (Leica biosystems) and frozen sections were stained with antibodies against NPRA (Fabgennix), CD31 (R&D), phosphorylated endothelial nitric oxide synthase (p‐eNOS; Abcam). Secondary antibodies were conjugated with Cy3 (Biolegend). The aorta sections were counterstained with Hoechst 33258 (Beyotime). Photographs were captured under confocal microscope LSM800 (Zeiss).

### 4.14 Reactive oxygen species (ROS) measurement

The cells were incubated with 10 μmol/L of ROS detection probe DCFH‐DA (SigmaAldrich) at 37°C for 30 min and then digested with trypsin without EDTA. After centrifugation at 1000 *g* for 3 min and three washes with PBS, the cells were injected into FACSVerse flow cytometer (BD Bioscience) within 1 h. The data were evaluated through flowjo v10 software (BD Bioscience).

The mouse thoracic aorta were frozen sectioned at 5 μm in thickness and fixed with 4% paraformaldehyde for 10 min. The sections were stained with 10 μmol/L of Dihydroethidium (DHE; Beyotime) for 30 min. The results were observed under confocal microscope LSM800 (Zeiss).

### 4.15 Nitric oxide (NO) measurement

The serum from mice were collected. The levels of NO were examined using a commercial nitrite detection kit (Beyotime) according to the manufacturer Manual. Briefly, the sample (50 μl) was mixed with 50 μl of Griess Reagent I and Griess Reagent II at room temperature. The absorbance was detected at 540 nm.

### 4.16 Statistical analysis

All data are shown as means ± SD. Statistical analysis was conducted using the GraphPad Prism software. Two‐tailed Student's *t‐*test was used for the comparison between two groups for gene expression in HUVECs between two PDLs and mice at two different ages. One‐way ANOVA followed by Tukey's post hoc test was carried out for comparing with three or more groups for vasorelaxation in mice by different dosages. Two‐way ANOVA was performed for multiple comparison with two variables (genotype vs treatment or between two treatments) for the expression and cell cycle progression in HUVECs or in mice. A probability value of <0.05 was considered as statistical significance.

## AUTHOR CONTRIBUTIONS

CL, SC, and XLT conceived and designed the study. CL, HL, WZ, and LC performed the experiments, data processing, and analysis. ZY and YX involved in discussion and provided academic input. ST performed English editing for the manuscript. CL and SC prepared manuscript. XLT and SC conducted supervision, manuscript drafting, revising, and editing. All authors critically read and commented on the manuscript.

## CONFLICT OF INTEREST

The authors declare no conflict of interest.

## Supporting information


TableS1

## Data Availability

All data generated and/or analyzed during this study are included in this article, and the data that support the results of this study are available from the corresponding author upon reasonable request.
