# Supplementary material for: Chronological attenuation of NPRA/PKG/AMPK signaling promotes vascular aging and elevates blood pressure
Source: Aging Cell. 2022 Aug 25;21(9):e13699. doi: 10.1111/acel.13699 (PMC9470896; doi:10.1111/acel.13699)
Supplement: Supplementary file 1 — TableS1 [file ACEL-21-e13699-s001.docx]

**Supplementary Table 1. Primer sequences used for qPCR**

| Primer name | Primer sequence (5’-3’) |
| --- | --- |
| Homo-NPRA-F | TGAGCCCAGTAGCCTTGAGA |
| Homo-NPRA-R | CTCAATGCGTTTACGGTTCA |
| Homo-P21-F | TAGCAGCGGAACAAGGAG |
| Homo-P21-R | AAACGGGAACCAGGACAC |
| Homo-IL6-F | GTCAGGGGTGGTTATTGC |
| Homo-IL6-R | TCATCACTGGTCTTTTGGAG |
| Homo-IL8-F | ACTTCTCCACAACCCTCTG |
| Homo-IL8-R | TACTCCAAACCTTTCCACC |
| Homo-β-actin-F | GCCGACAGGATGCAGAAGGAGATCA |
| Homo-β-actin-R | AAGCATTTGCGGTGGACGATGGA |
| TELG | ACACTAAGGTTTGGGTTTGGGTTTGGGTTTGGGTTAGTGT |
| TELC | TGTTAGGTATCCCTATCCCTATCCCTATCCCTATCCCTAACA |
| HBGU | CGGCGGCGGGCGGCGCGGGCTGGGCGGCTTCATCCACGTTCACCTTG |
| HBGC | GCCCGGCCCGCCGCGCCCGTCCCGCCGGAGGAGAAGTCTGCCGTT |
